# Supplementary material for: M3C: Monte Carlo reference-based consensus clustering
Source: Sci Rep. 2020 Feb 4;10:1816. doi: 10.1038/s41598-020-58766-1 (PMC7000518; doi:10.1038/s41598-020-58766-1)
Supplement: Supplementary file 1 — Supplementary Information. [file 41598_2020_58766_MOESM1_ESM.pdf]

Supplementary materials for

M3C: Monte Carlo reference-based consensus clustering

Christopher R. John, David Watson, Myles Lewis, Dominic Russ, Katriona Goldmann, Michael Ehrenstein, Costantino Pitzalis, Michael Barnes

Supplementary figure 1

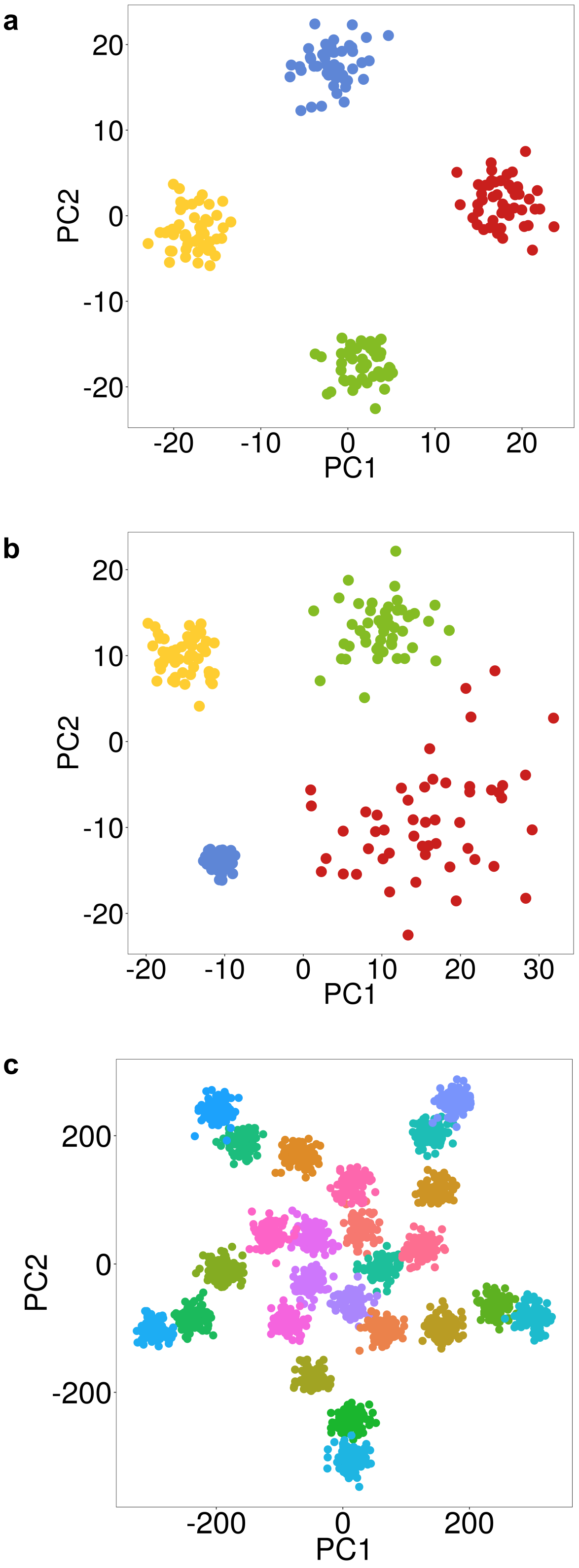

Supplementary figure 1. Clusterlab demonstration of the simulation of three synthetic datasets. For explanation of parameters see methods. (A) Simulation of 4 Gaussian clusters with $D = 1$, $M = 50$, $r = 8$. (B) Simulation of 4 Gaussian clusters with $D = (0.5,1,1.5,3)$, $M = 50$, $r = 8$. (C) Simulation of 5 rings of 5 Gaussian clusters with $D = 6$, $M = 100$, $r = 7$, $\beta = (2,4,6,8,10)$, $\theta = (30,90,180,0,0)$.

Supplementary figure 2

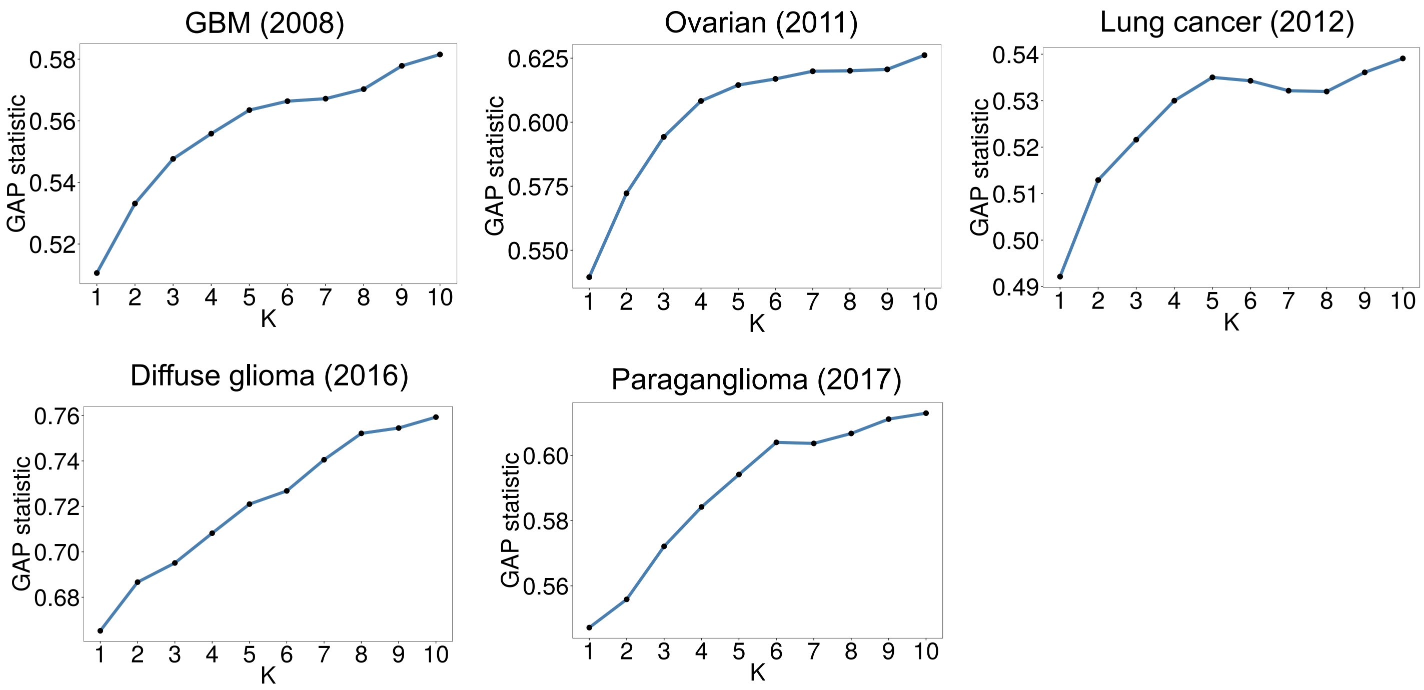

Supplementary figure 2. Results from running the GAP-statistic across 5 datasets using the PAM algorithm. A potentially misleading trend towards increased stability at higher values of K can clearly be observed.

Supplemental figure 3

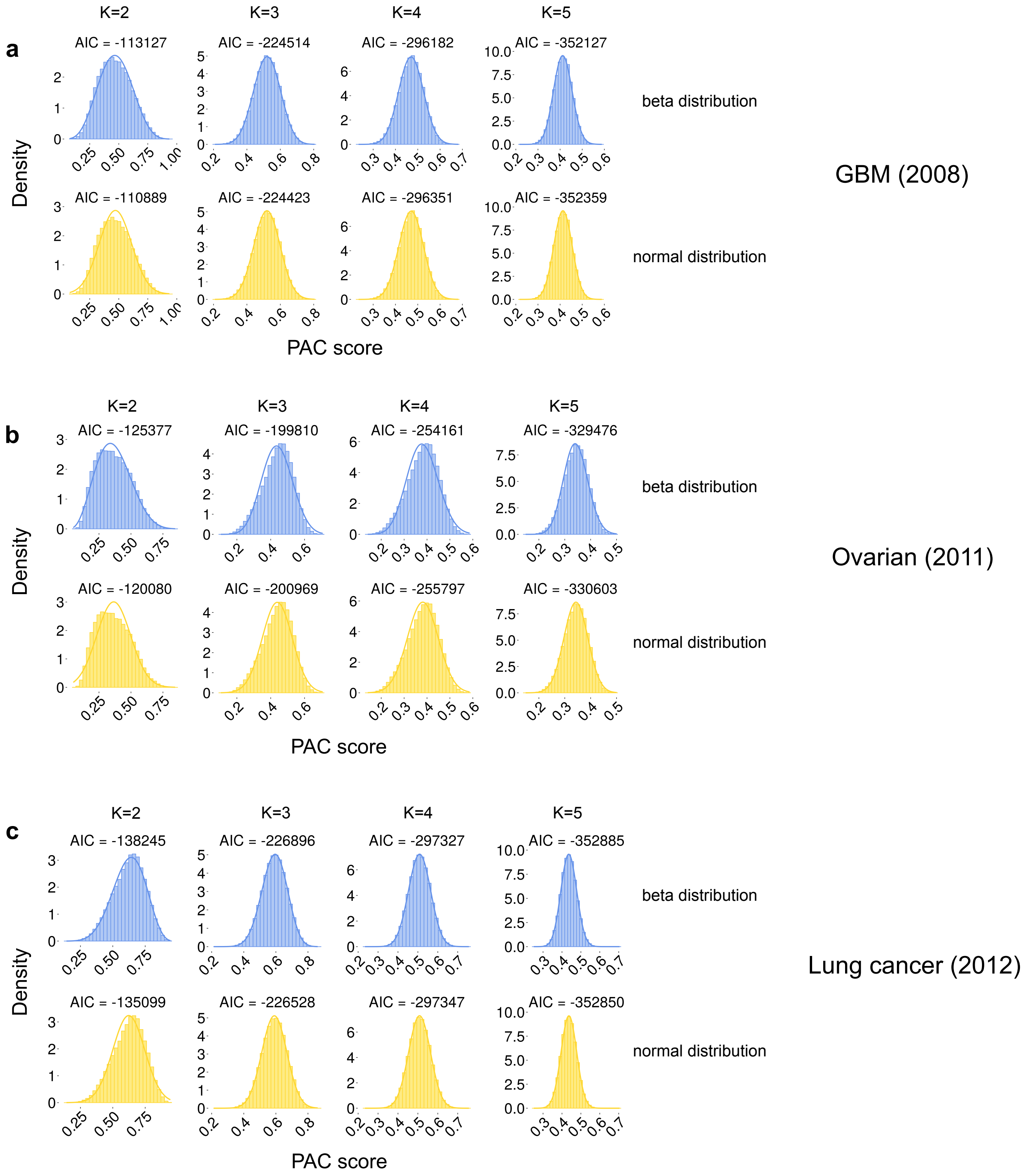

Supplementary figure 3. Reference distributions generated using M3C of PAC scores across the range of K from 2-5. 100,000 simulations were performed to generate the distributions and a normal (yellow) and beta (blue) fit were compared. Using the Akaike information criterion (AIC) it can be observed that for K=2, a beta distribution fits slightly better than the normal (indicated by a more negative AIC). This is due to the skew and kurtosis that the reference distributions often have at K=2.

Supplemental figure 4

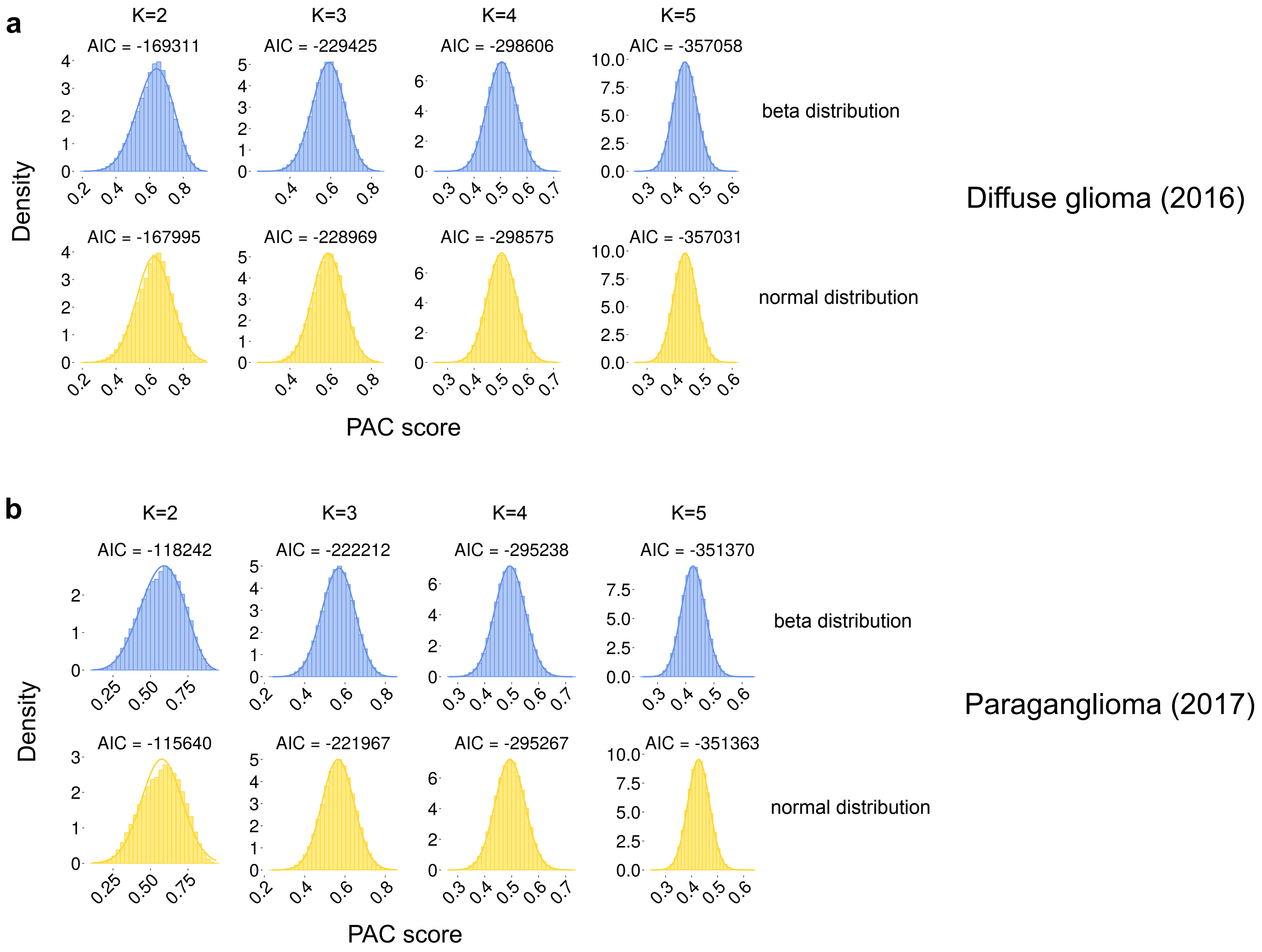

Supplementary figure 4. See supplementary figure 3 legend for details, the same analysis was performed, but with different datasets.

Supplementary figure 5

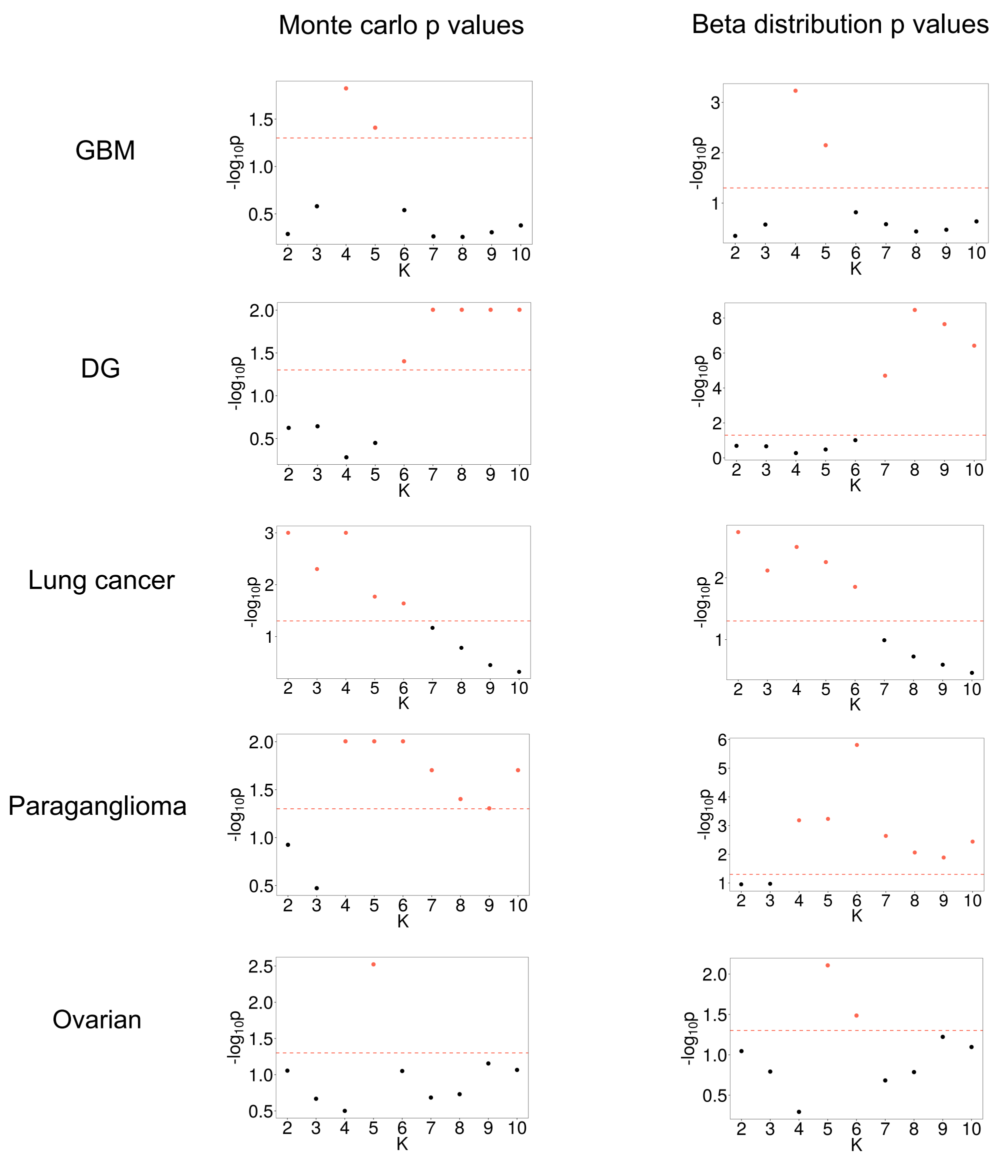

Supplementary figure 5. Comparing M3C Monte Carlo p values with p values estimated from a beta distribution. The beta distribution is generated using parameters from the initial N Monte Carlo simulations M3C performs. Tail estimation allows the limits of a finite number of simulations to be overcome.

Supplementary figure 6

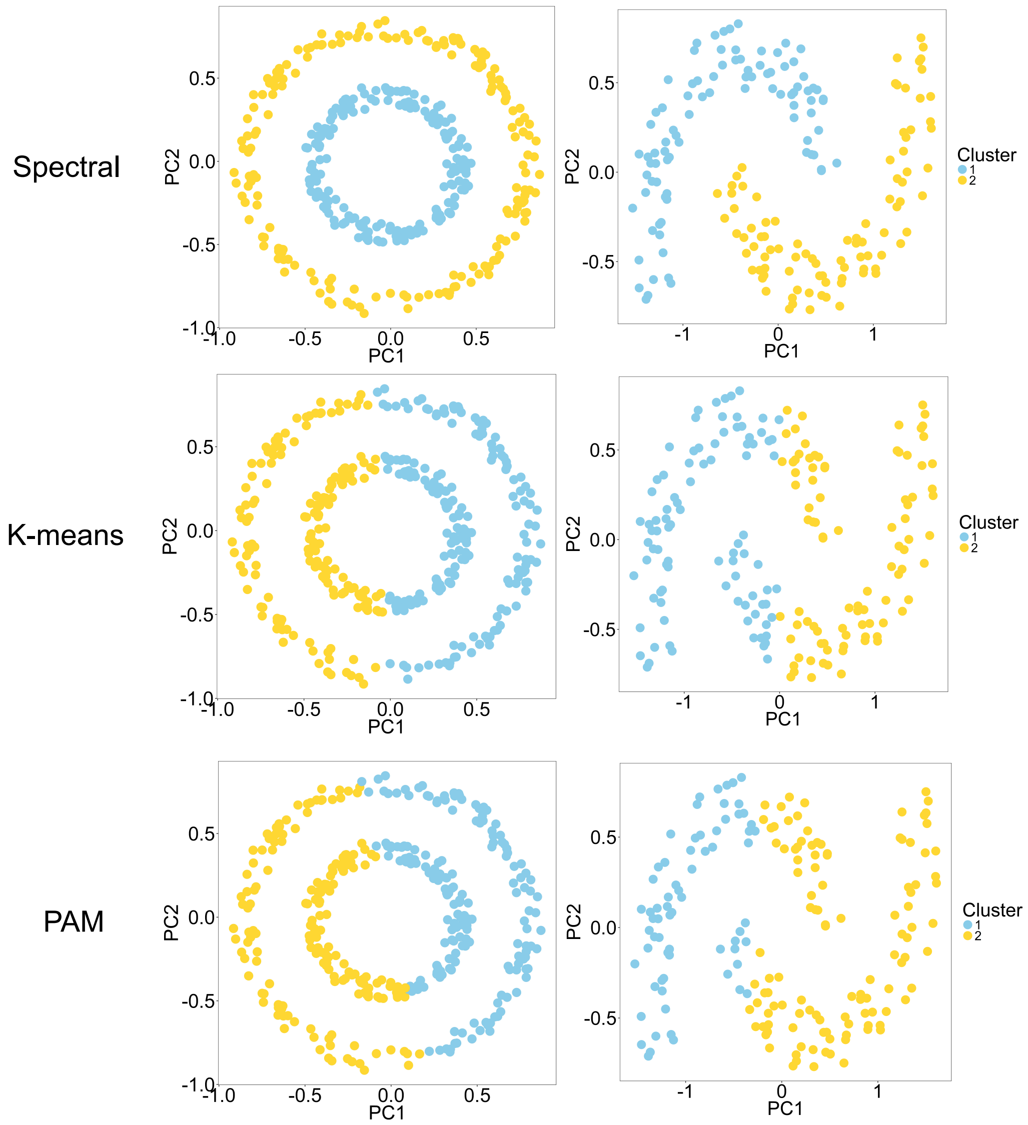

Supplementary figure 6. Comparing the M3C results using spectral clustering, PAM, or K-means on concentric circles or half-moon shapes.

Supplementary figure 7

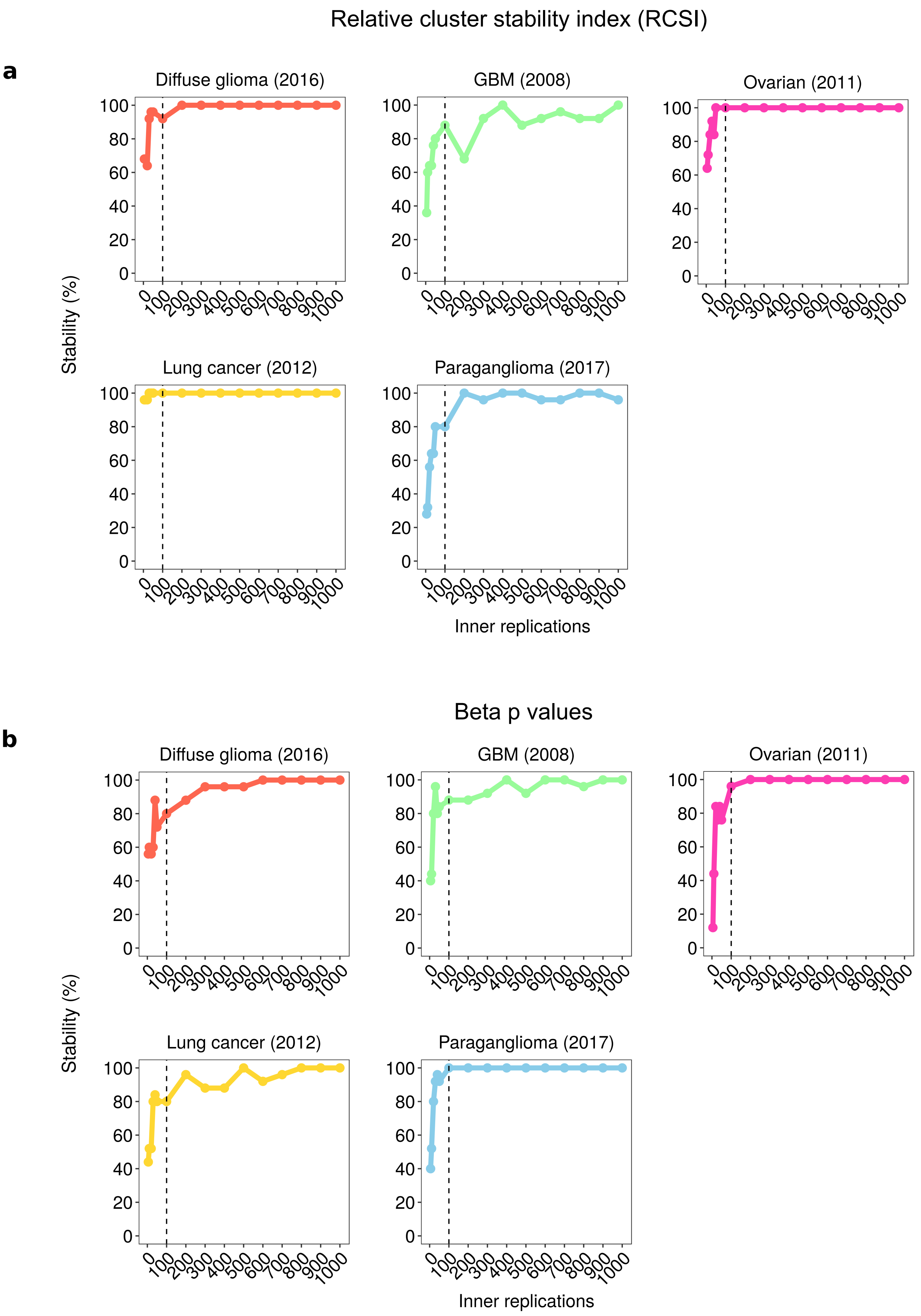

Supplementary figure 7. A sensitivity analysis of M3C parameters across 5 real datasets. (A) Results from the RCSI are shown. The inner resampling replications of M3C were varied, while the outer Monte Carlo simulations were left to default (value of 100). Stability is measured on the y axis as the percentage of times the same result was obtained across 25 iterations. (B) Results from the beta distribution after conducting the same analysis described in (A).

Supplementary figure 8

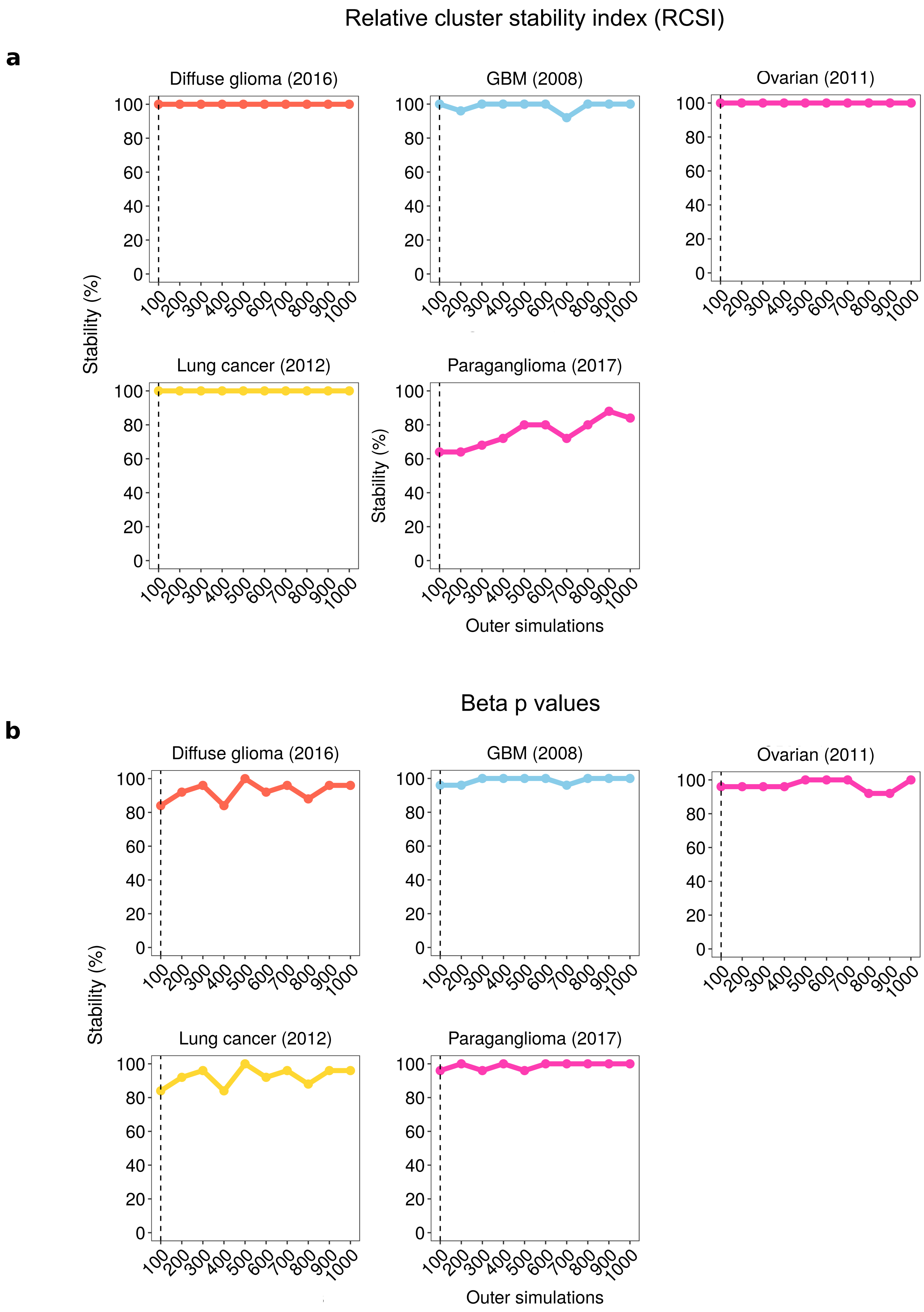

Supplementary figure 8. Same analysis described in supplementary figure 7, except the outer Monte Carlo simulations of M3C were varied, the inner resampling replications were left to default (value of 100).

Supplementary Table I: More detailed information about datasets selected for analysis. HC refers to hierarchical clustering.

**Publication**	**Year**	**Data type**	**Original Algorithm**	**Original score metric**	**Number of features**	**Data format**	**Samples**	**M3C K**	**Original K**
Glioblastoma	2008	Microarray	Consensus clustering (HC)	Not stated	1740	log2(norm)	206	4	4
Ovarian Carcinoma	2011	Microarray	NMF Consensus clustering	Cophenetic	800	log2(norm)	489	5	4
Squamous cell lung cancers	2012	RNA-seq	Consensus clustering (HC)	Not stated	2257	log2(RPKM)	178	2	4
Human breast tumors	2012	miRNA-seq	NMF Consensus clustering	Cophenetic	306	log2(CPM)	697	1	7
Diffuse Glioma	2016	RNA-seq	Consensus clustering (HC)	Calinsky-Harabasz	2275	log2(CPM)	667	8	4
Lupus	2016	Microarray	HC	None	48	log2(norm)	82	1	7
Pheochromocytoma and Paraganglioma	2017	RNA-seq	Consensus clustering (HC)	Not stated	3000	log2(TPMs)	173	6	4

Supplementary note 1

M3C pseudo code

INPUTS: T = Data

PARAMS: B = Monte Carlo simulations, H = Inner resampling iterations, K = maximum K, clustering algorithm = PAM (default)

REFERENCE

FOR $b = 1 \dots B$:

    Calculate random data Q^b (equations 1-3)

    Calculate Euclidean distance matrix

    FOR $h = 1 \dots H$:

        Resample Q^b

        Create or update the indicator matrix I (equation 4)

        FOR $k = 1 \dots K$:

            Cluster distance matrix using clustering algorithm

            Assign clustering to connectivity matrix M (equation 5)

    Normalise M (equation 6)

    Calculate CDF and reference PAC scores (equations 7-8)

REAL DATA

Calculate Euclidean distance matrix

FOR $h = 1 \dots H$:

    Resample T

    Create or update the indicator matrix I (equation 4)

    FOR $k = 1 \dots K$:

        Cluster distance matrix using inner clustering algorithm

        Assign clustering to connectivity matrix M (equation 5)

Normalise M for $k = 1 \dots K$ (equation 6)

Calculate CDF then get real PAC score for each k (equations 7-8)

FINAL STEPS

Calculate RCSI for $k = 1 \dots K$ (equation 9)

Calculate p values for $k = 1 \dots K$ (equation 10)
